# The use of single-timepoint images to link administered radioiodine activity (MBq) to a prescribed lesion radiation-absorbed dose (cGy): a regression-based prediction interval tool for the management of well-differentiated thyroid cancer patients

**DOI:** 10.1007/s00259-023-06240-1

**Published:** 2023-05-12

**Authors:** Audrey Mauguen, Ravinder K. Grewal, Finn Augensen, Murad Abusamra, Sonia Mahajan, Vetri Sudar Jayaprakasam, Joseph Osborne, Sofia Haque, Bernadette Z. Y. Wong, Ronald A. Ghossein, James Fagin, Heiko Schӧder, R. Michael Tuttle, Alan Ho, John L. Humm, Steven M. Larson

**Affiliations:** 1 Department of Epidemiology & Biostatistics, Memorial Sloan Kettering Cancer Center, New York, NY USA; 2 Department of Radiology, Memorial Sloan Kettering Cancer Center, 415 East 68th Street, Z-2064, New York, NY 10065 USA; 3 Department of Medical Physics, Memorial Sloan Kettering Cancer Center, New York, NY USA; 4 Division of Molecular Imaging and Therapeutics, Weill Cornell Medical College, New York, NY USA; 5 Department of Pathology and Laboratory Medicine, Memorial Sloan Kettering Cancer Center, New York, NY USA; 6 Department of Medicine, Memorial Sloan Kettering Cancer Center, New York, NY USA

**Keywords:** Differentiated thyroid cancer, Dosimetry, Iodine-124, PET/CT, Radioactive iodine therapy

## Abstract

**Purpose:**

To introduce a biomarker-based dosimetry method for the rational selection of a treatment activity for patients undergoing radioactive iodine ^131^I therapy (RAI) for metastatic differentiated thyroid cancer (mDTC) based on single-timepoint imaging of individual lesion uptake by ^124^I PET.

**Methods:**

Patients referred for RAI therapy of mDTC were enrolled in institutionally approved protocols. A total of 208 mDTC lesions (in 21 patients) with SUV_max_ > 1 underwent quantitative PET scans at 24, 48, 72, and 120 h post-administration of 222 MBq of theranostic NaI-^124^I to determine the individual lesion radiation-absorbed dose. Using a general estimating equation, a prediction curve for biomarker development was generated in the form of a best-fit regression line and 95% prediction interval, correlating individual predicted lesion radiation dose metrics, with candidate biomarkers (“predictors”) such as SUV_max_ and activity in microcurie per gram, from a single imaging timepoint.

**Results:**

In the 169 lesions (in 15 patients) that received ^131^I therapy, individual lesion cGy varied over 3 logs with a median of 22,000 cGy, confirming wide heterogeneity of lesion radiation dose. Initial findings from the prediction curve on all 208 lesions confirmed that a 48-h SUV_max_ was the best predictor of lesion radiation dose and permitted calculation of the ^131^I activity required to achieve a lesional threshold radiation dose (2000 cGy) within defined confidence intervals.

**Conclusions:**

Based on MIRD lesion-absorbed dose estimates and regression statistics, we report on the feasibility of a new single-timepoint ^124^I-PET-based dosimetry biomarker for RAI in patients with mDTC. The approach provides clinicians with a tool to select personalized (precision) therapeutic administration of radioactivity (MBq) to achieve a desired target lesion-absorbed dose (cGy) for selected index lesions based on a single 48-h measurement ^124^I-PET image, provided the selected activity does not exceed the maximum tolerated activity (MTA) of < 2 Gy to blood, as is standard of care at Memorial Sloan Kettering Cancer Center.

**Trial registration:**

NCT04462471, Registered July 8, 2020.

NCT03647358, Registered Aug 27, 2018.

**Supplementary Information:**

The online version contains supplementary material available at 10.1007/s00259-023-06240-1.

## Introduction

Precision medicine strives to tailor the best possible treatment to the unique cancer of an individual patient. Distant metastases are detected in 3–20% of patients with differentiated thyroid cancer (DTC) at some point in the course of their disease [1]. For advanced thyroid cancer, treatment of metastatic DTC (mDTC) with radioiodine ^131^I therapy (RAI) has been lifesaving for many patients [2]. However, not all patients benefit, and side effects can be significant. It is known that response of thyroid cancer to RAI is radiation dose-related, but unlike modern external beam radiotherapy, there are no widely accepted dosimetry methods to predict which patients with metastatic thyroid cancer are likely to respond to RAI therapy. Accordingly, many patients continue to receive multiple empirical therapeutic doses of ^131^I that may be ineffective and can cause considerable morbidity, with potential toxicity to the bone marrow, lung, and salivary glands. In this paper, we propose a statistical approach that equips the nuclear medicine physician with a tool to select an appropriate index lesion and provide activity that needs to be administered to achieve a desired radiation-absorbed dose based on a single 48-h PET SUV from an ^124^I PET scan. In patients with multiple lesions, this could be the hottest, coldest, or intermediate lesion depending on type of treatment. SUV_max_ was the parameter of choice for our analysis, because ~ 80% of the metastatic lesions were small (< 1 cm), close to the limits of resolution of the PET scanner.

Several investigators have proposed the use of ^124^I as a theranostic solution to the problem of RAI dosimetry [3-9]. Iodine-124 is a 4.2-day half-life positron-emitting isotope that allows for serial PET imaging over several days, enabling accurate lesion dosimetry using the MIRD approach [10]. A simple correction for the physical half-life and emissions between imaging isotope ^124^I and therapeutic isotope ^131^I provides the capability to predict the lesion doses from a planned ^131^I therapy administration. Such radionuclide dosimetry may allow nuclear medicine physicians and referring physicians alike to better identify patients likely to benefit from RAI and those who will not, thereby preventing unnecessary treatment when the predicted tumor doses are below the levels required to achieve therapeutic responses.

A common clinical problem is that some patients are refractory to ^131^I RAI (RAIR); this resistance is most often because their tumors do not concentrate and retain sufficient RAI to be tumoricidal. Our interest in ^124^I was intensified based on the discoveries of Fagin et al. [11], who demonstrated that kinase inhibitors of the MAP-kinase pathway, particularly MEK and BRAF inhibitors, could reinduce RAI tumor uptake in laboratory models of BRAF-mutant thyroid cancer [12]. Accordingly, we investigated the potential of a single-timepoint dosimetry method using PET/CT ^124^I imaging, based on what we called the 48/48-h rule (48-h timepoint, 48-h effective half-life). In a group of patients with RAIR thyroid cancer studied by Ho et al. [12], we found that single-timepoint quantitative PET imaging at 48 h could successfully be used to select patients for RAI therapy. An increase in radioiodine uptake induced by a 4-week course of a kinase inhibitor (determined by ^124^I-uptake with PET imaging performed at 48 h) that predicted a radiation-absorbed dose greater than 2000 centiGray (cGy) correlated with a partial response 6 months post-RAI per RECIST criteria in 5/8 patients [12].

In the present study, we introduce a regression-based RAI dosimetry tool for mDTC with a known precision that builds on these earlier findings in the RAIR setting. Our hypothesis was that a practical and clinically useful dosimetry biomarker could be developed using single-timepoint ^124^I PET imaging, to (1) reliably predict ^131^I RAI lesion radiation-absorbed dose for all active lesions in an individual patient; and (2) optimize selection of administered activity (MBq) necessary to achieve at least the minimum radiation dose needed to reliably induce a treatment effect. If successful, this approach could offer practical guidance for selecting treatment activities for patients with heterogeneous radioiodine uptake that would produce lesion doses within an expected statistical prediction interval.

In this manuscript, we discuss the workflow based on serial quantitative ^124^I PET imaging with dosimetry estimates derived from the lesion uptake and clearance kinetics of individual lesions. Our approach sought to determine the best single-timepoint imaging and test its precision as a predictor of lesion dosimetry, minimizing the need for four-timepoint data acquisition. Our main motivation for developing the ^124^I PET imaging biomarker approach was to devise a practical and simple methodology to determine lesional dosimetry that could be combined with standard blood and whole-body clearance dosimetry to optimize ^131^I RAI recommendations for patients with mDTC.

## Methods

### Population

This study includes lesions from consecutive patients studied at MSK under two different IRB-approved protocols, 18–253 and 20–053, who underwent imaging between March 2019 and August 2021. Patients who were considered candidates for RAI treatment of DTC were enrolled after giving informed consent. All patients had histologically confirmed metastatic thyroid cancer (Table 1). The tumors were histologically classified according to the most recent World Health Organization (WHO) classification of thyroid tumors [13]. High-grade follicular cell-derived non-anaplastic thyroid carcinomas were then assigned a papillary or follicular phenotype on the basis of the presence or absence of the nuclear features of papillary thyroid carcinoma.

**Table 1:** Patient demographics

Characteristic	*N* = 21*
Age (years)	57 [22–85]
Sex	
Female	8 (38%)
Male	13 (62%)
Stage	
Stage IV	21 (100%)
Histology	
Papillary	18 (86%)
Follicular	3 (14%)
Thyroglobulin	153 [0–139, 750]
Lesions per patient	11 [3–23]
Presence of lung nodules	17 (81%)
Presence of bone metastases	8 (38%)
Presence of neck nodes	5 (24%)
Presence of thoracic nodes	9 (43%)
Presence of muscle/soft tissue nodes	3 (14%)
Presence of thyroid bed nodes	8 (38%)
Presence of liver metastases	4 (19%)
Maximum tolerated activity (MTA) mean	15.76 GBq (426 mCi)
MTA range	1.74–33.15 GBq (47–896 mCi)
Received treatment	15 (71%)
Mean ^131^I activity given to patients	7.18 GBq (194 mCi)
Range of ^131^I activities given to patients	1.70–15.06 GBq (46–407 mCi)

*Median [range]; *n* (%)

*mCi*, milliCurie

### Individualized lesion kinetics and dosimetry

**Fig. 1:**
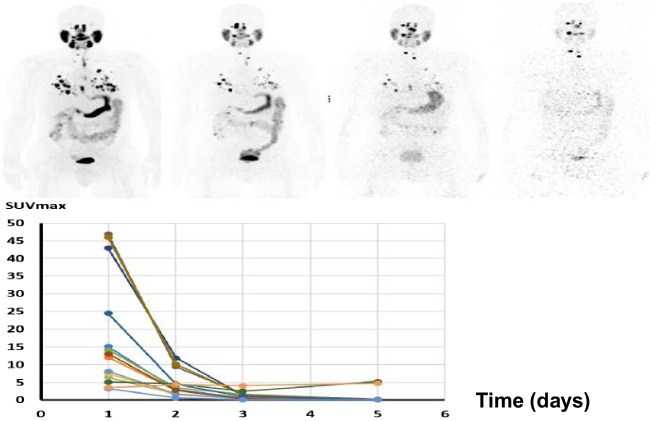
Example of four ^124^I PET scans conducted at 24, 48, 72, and 120 h post-oral radioiodine administration. The clearance curves (SUV_max_ plotted vs. time in days) for individual neck and lung lesions of size > 0.5 cc) are shown in the view graph. This patient has lung lesions exhibiting high radioiodine uptake and rapid clearance accompanied by neck nodes with low uptake and slow clearance. This is an example of a patient who was not selected for treatment, since overall dose for several lesions was well below the 2000-cGy threshold

Lesions were scanned at four timepoints by PET/CT after oral administration of a diagnostic activity 222 MBq** (**6.0 mCi) of ^124^I-NaI (3D Imaging, Waco, TX). Our selection of 222 MBq was made for three reasons: (i) to account for the low positron yield of ^124^I (only 0.23), (ii) because of our need to characterize the pharmacokinetics of lesion uptake (including the smallest identifiable lesion) out to 5 days post-administration, and (iii) we knew that most of these patients would undergo ^131^I radioiodine therapy with high administered activities. Whole-body imaging was performed on a GE D710 PET/CT 3-ring scanner with an axial field-of-view of 15.3 cm at the nominal times: 24, 48, and 72, 120 h post-administration (Fig. 1). The number of minutes per bed position was determined so that the whole-body scan duration from the vertex to mid-thigh would be ~ 60 min, i.e., between 6 and 8 min. ^124^I PET reconstructions were performed in a 128 * 128 matrix, 2 iterations, 16 subsets, with in-plane smoothing with a 6.4-mm FWHM Gaussian kernel, and GE *z*-axis heavy smoothing with the prompt gamma correction turned on. Regions of interest (ROI) were placed over all visible lesions > 0.5 cm within the body using ^124^I PET/CT and the diagnostic CT images. From each ROI, the following parameters were recorded in an Excel database for each patient and each lesion: size in three dimensions (cm); the maximum standardized uptake value (SUV_max_) by weight and by lean body weight, and activity concentration in MBq/gram or microcurie/gram (Table 2). For these patients, we estimated the best clearance fitting curve using a three-parameter (a_0_, *λ*_1_ and *λ*_2_) dual exponential equation model comprising lesion uptake a_0_(1-exp^−(^*λ*_1_^*t*)^) and clearance (exp^−(^*λ*_2_^*t*)^). Prior to integration, clearance fitting was adjusted to replace the decay constant of ^124^I with ^131^I, used for therapy. This area under the curve (AUC) of the activity per gram (MBq.hr/g) was multiplied by the equilibrium dose constant (10.95 g.cGy/MBq·h or 0.405 g.cGy/µCi·h) for the non-penetrating β-emissions of ^131^I to yield the lesion-absorbed dose in cGy. The AUC is an integrated measure over time of the kinetics of uptake and clearance, which determines how much radiation is retained within an individual lesion during RAI treatment. Partial volume corrections were performed based on the theoretical curve derived for a scanner with a full width at half maximum (FWHM) of 6 mm published in the paper by Soret, Bacharach, and Buvat [14].

**Table 2:** Abbreviated table of parameters determined for each lesion with measured ^124^I radioiodine uptake from patient #1. The maximum tolerated activity (MTA) for this patient was 19.72 GBq (533 mCi) but the patient was administered 15.06 GBq (407 mCi) of ^131^I

Lesions	Mean size (cm)	Lesion dose (non-PVC) in cGy/GBq	Lesion dose (PVC) in cGy/GBq	T1/2 effective (days)	AUC (uCi h/g)	uCi/g at 48 h	SUV_max_ (24 h)	SUV_max_ (48 h)	SUV_max_ (72 h)	Activity in GBq (mCi) to deliver 2000 cGy	Absorbed dose (cGy) from administered 19.72-GBq treatment	Absorbed dose (cGy) at MTA
R. parietal skull	4.03	868	1133	8.02	79.28	0.29	21.9	25.58	25.31	1.77 (47.7)	17,063	22,345
L. scapula	3.63	557	749	8.02	50.87	0.20	14.66	17.31	17.55	2.67 (72.1)	11,284	14,778
R. ant. 2 rib	4.10	440	573	8.02	40.22	0.15	10.89	13.85	13.18	3.49 (94.4)	8622	11,291
L. lateral 7 rib	3.17	490	695	8.02	44.73	0.17	12.79	14.65	16.07	2.88 (77.8)	10,469	13,711
L. post elements T3	1.07	68	240	2.33	6.17	0.05	5.54	3.57	3.52	8.33 (225.0)	3618	4739
spinous process T4	0.77	247	1398	8.02	22.52	0.09	6.56	7.68	8.58	1.43 (38.7)	21,056	27,575
T7 vertebral body	0.60	215	1920	5.24	19.66	0.10	8.83	8.03	7.68	1.04 (28.1)	28,916	37,869
L2 vertebral body	0.40	193	2703	4.90	17.63	0.09	7.71	8.26	7.56	0.74 (20.0)	40,710	53,314
L. post. acetabulum	4.27	213	275	6.30	19.46	0.09	7.32	6.87	7.07	7.29 (196.9)	4134	5414
L. post. 5 rib	1.90	49	97	8.02	4.51	0.02	1.63	1.24	1.25	20.59 (556.4)	1462	1916
L3 vertebral body	0.87	19	90	2.21	1.76	0.01	1.48	1.35	0.84	22.19 (599.7)	1357	1778
Ant. aspect of thyroid cartilage	2.00	2260	6348	8.02	306.97	1.26	95.8	127.01	84.06	0.31 (8.5)	95,597	125,193
L. thyroid bed	0.93	203	865	6.75	18.57	0.08	5.44	7.7	5.31	2.31 (62.5)	13,022	17,054

*cm*, centimeters; *PVC*, partial volume correction; *cGy*, centiGray; GBq, GigaBecquerel, *T1/2*, half-life; *AUC*, area under the curve; *h*, hour; *uCi/g*, microcurie/gram; *mCi*, millicurie; *R*, right; *L*, left; *Ant*, anterior; *Post*, posterior

Columns 6 and 7 can be converted to MBq h and MBq/g by multiplying by 0.037.

### Predicting AUC based on a single timepoint

A goal of this study is to develop a statistical model to predict the calculated lesion dosimetry derived from four-timepoint imaging with fitted radioiodine kinetics from a single image acquisition timepoint. If successful, this would obviate the need for costly additional PET/CT imaging needed to fully characterize the kinetic behavior. The approach starts with estimating the linear relationship between the full dosimetry derived from the AUC from fitting four measured timepoints and the activity measured at one timepoint, called the predictor (e.g., SUV_max_ at 48 h). The absorbed dose for an individual lesion is directly proportional to AUC by a multiplicative factor, the equilibrium dose constant, which describes the emission properties of the radionuclide, and is inversely proportional to lesion mass (note that for PET scanners the voxel values are already in units of activity per unit mass; i.e., Bq/g). For this estimation, the unit is the lesion, and a generalized estimating equation approach is used to estimate the parameters (intercept, slope, and robust variance matrix) accounting for the correlation between lesions in the same patient. Log-transformed values of the uptake and doses are used to ensure the data are normally distributed. The linear model is as follows, where the errors ${\varepsilon }_{ij}$ are correlated, ${y}_{ij}$ is the logarithm of the AUC value, and ${x}_{ij}$ is the uptake measured at one timepoint and ${x}_{ij}^{^{\prime}}$ is the transpose of the matrix ${x}_{ij}$; e.g., the logarithm of a 48-h SUV_max_ measured for lesion *j* from patient *i*:

1 $${y}_{ij}={x}_{ij}^{^{\prime}}\beta +{\varepsilon }_{ij}$$

Second, using the estimations for $\beta$ and the covariance matrix, a prediction interval (PI) is calculated. A PI differs from a confidence interval, as it aims to predict with 95% confidence where future measurements will fall. In our case, if we observed the same value of SUV_max_ at 48 h for 100 new lesions, the PI is the range in which 95 of those lesions’ AUCs will be found. As difficulties arose when analytically constructing the PI, we used simulated prediction to calculate PIs following the steps detailed in Gelman and Hill [15] and summarized in Appendix A.

To validate the accuracy of the prediction, we used a leave-one-patient-out cross-validation approach. For each patient $i (i=1,\dots ,n)$, the linear regression parameters are re-estimated using $n-1$ patients (excluding $i$), and PIs are calculated for each lesion based on their measured predictor values. For those lesions, the actual observed AUC is then compared to the PI. When using 95% PI, it is expected that 95% of the observed values will fall into the corresponding PIs; i.e., 5% will be outside the prediction. In addition, for each left-out patient, an error of prediction is calculated corresponding to the squared difference between the predicted and true AUC values for each lesion. This squared error is the average over all the lesions of all the patients to obtain a cross-validated error.

### Range of ^131^I activity to treat a chosen efficacy rate for RAI in mDTC patients

The minimum acceptable target radiation dose of 2000 cGy was chosen because doses above this level are often used as the threshold for lesions to receive radioiodine treatment [16]. Based on the PI available for the AUC, a simple calculation of the relationship between ^124^ and ^131^I uptake can yield a PI for the dose [*d*_low_ − *d*_high_]. For a 95% PI, the interval shows the ^131^I activity that will ensure a dose of 2000 cGy in 95% of the lesions with the corresponding measured uptake. Thus, the higher boundary (*d*_high_) corresponds to the activity to deliver at least 2000 cGy to 97.5% of the lesions with the given uptake. By varying this boundary, it is possible to select an activity that will target 95%, 90%, or fewer of the lesions. This provides the treating physician with information necessary to select a balance between the activity needed and the predicted efficacy.

### Memorial Sloan Kettering maximum tolerated activity

Since 1962, MSK clinicians have employed a series of simple blood and whole-body clearance dosimetry benchmarks that provided guidelines for maximum tolerated activity (MTA) [17]. These guidelines have shown a remarkable safety record with respect to avoidance of serious toxicity to lung and bone marrow during high-dose RAI treatment for differentiated thyroid cancer. To perform these studies, serial blood samples and total body measurements are conducted to determine β and photon radiation dose contributions to blood (a surrogate for the dose-limiting bone marrow) from a pre-therapy tracer administration of ^131^I but which is readily adapted to ^124^I as in this study. The principal MTA guideline is that the radiation-absorbed dose to blood does not exceed 2 Gy. This MTA information provides the prescribing physician with an upper bound for the administered treatment activity of ^131^I, which can be used in combination with statistical lesion dose predictions to select the most appropriate treatment activity for that specific patient. The patients enrolled in this study were administered activities that did not exceed the maximum safe amount based on blood and whole-body clearance fitting as described by Furhang et al. [18].

## Results

### Patients

At present, we have analyzed data from 208 lesions in 21 individual patients. The median age was 57 years (range: 22–85) and 62% were male (Table 1). All had distant metastases. Patients had between 3 and 23 lesions (median = 11). From this cohort, 71% (15 patients, 169 lesions) were treated by ^131^I, with administered activities ranging from 1.70 to 15.06 GBq (46 to 407 mCi).

### Dosimetry

Each patient referred for RAI therapy with advanced mDTC undergoes pre-therapy dosimetry to determine MTA of ^131^I to avoid excessive radiation-absorbed dose to blood, lungs, and whole body during treatment. In the present study, we describe an extension of our dosimetry to include individual lesions. A full lesion dosimetric analysis based on the four ^124^I PET imaging timepoints was performed. An example of some of the data determined for patient #1 is shown in Table 2. The full results include, for each anatomical lesion site, the mean size (cm), lesion dose per projected unit GBq of administered ^131^I activity with and without partial volume correction [14], half-life based on exponential curve fitting, area under the curve based on an integrated curve fit, estimated activity per gram at 48 and 72 h post-administration, SUV and SUL (based on lean body mass) at 24, 48, and 72 h post-administration, the administered activity to deliver 2000 cGy, the radiation dose estimate for the administered therapy to the patient, and the maximum projected dose that could have been achieved had the maximum tolerated activity been administered. These results were used to derive a statistical model to predict the radiation dose to lesions. The dosimetry summary for all patients is given in Supplemental Table 1.

### Prediction of activity to deliver 2000 cGy

The prediction is limited to lesions with an SUV_max_ > 1, as no treatment is planned for lesions with no differential uptake. Lesion-absorbed doses were also calculated for lesions that received no treatment (n = 39) and included in the analysis, to include patients with low radioiodine avidity. For the dataset analyzed, the estimated regression coefficient (slope) is 1.002 (robust se = 0.024; 95% confidence interval: 0.954 to 1.049; *p* < 0.0001). The full predicted value of AUC based on 48 h uptake can be calculated as: $\widehat{\mathrm{AUC}}=\mathrm{exp}\left(0.697+1.002.\;\mathrm{ln}\;({\mathrm{SUVmax}}_{48})\right)$.

**Fig. 2:**
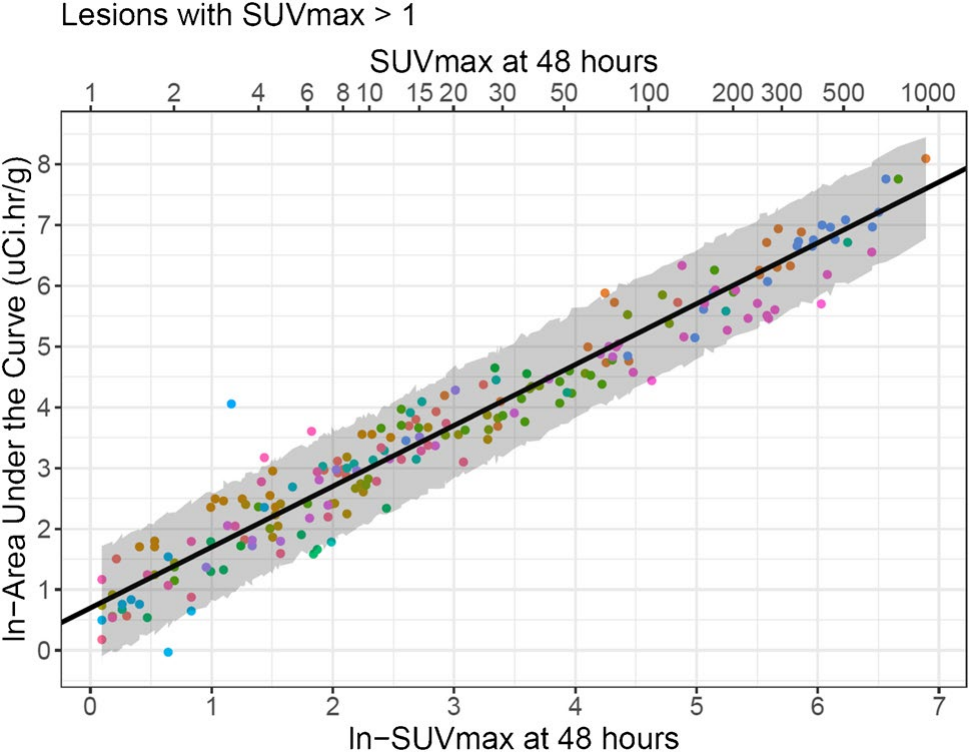
Prediction curve for the best predictor as the ^124^I PET imaging biomarker. ln-48-h SUV_max_ (optimal predictor) vs. ln-AUC (each color represents a patient; each dot is a lesion; the black line is the average linear regression line from the general estimating equation estimate while the gray area is the 95% prediction interval, which encompasses 95% of all lesions AUC at a particular 48-h SUV_max_, from the lowest value at the 2.5 percentile to the highest value at the 97.5 percentile). Because a logarithmic transformation is used, the distance between the average prediction and the measured values can be larger than they appeared

**Fig. 3:**
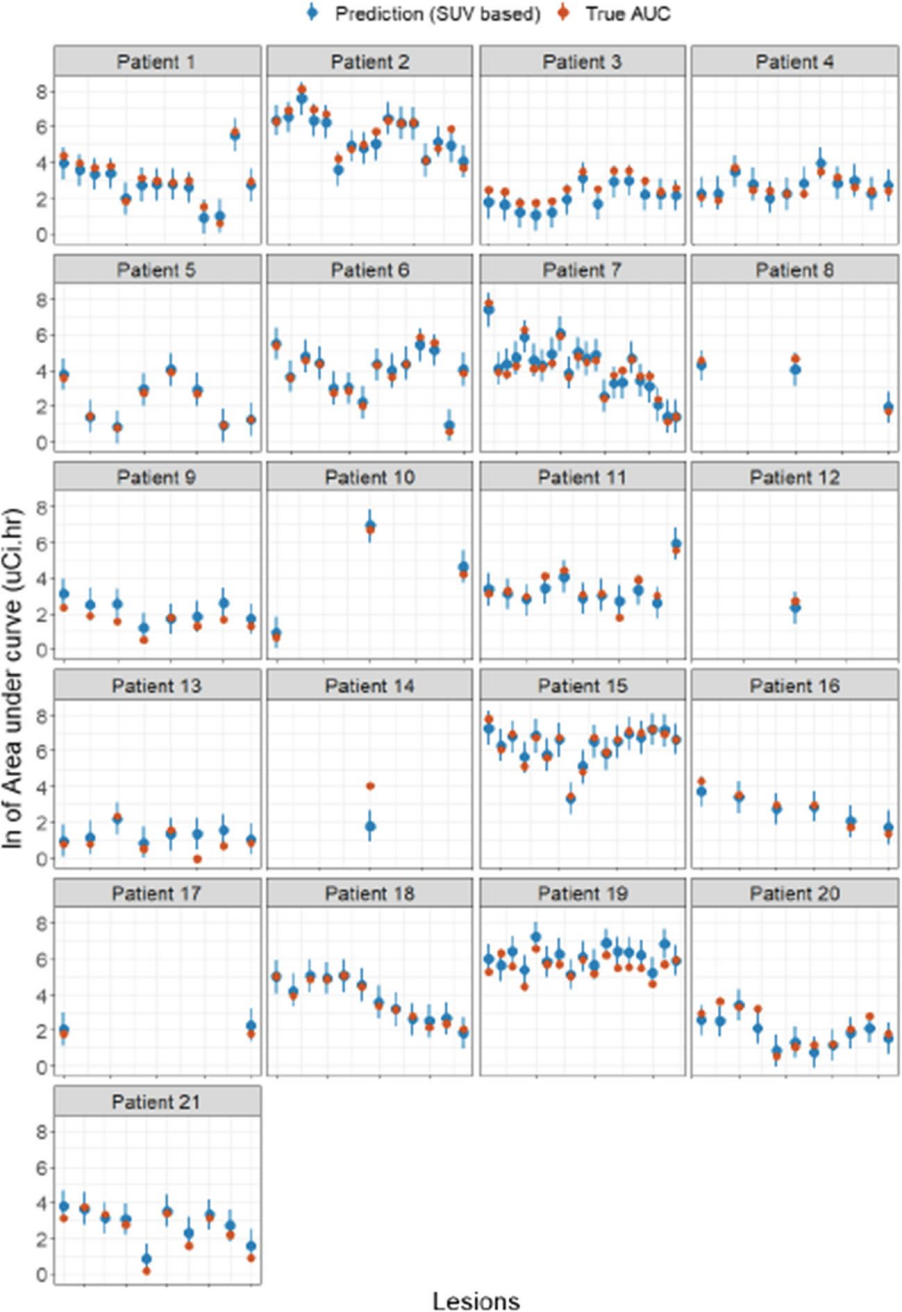
Results of leave-one-out cross-validation (SUV_max_ analysis). For each patient (separate quadrant), the AUC as predicted by our model is represented by a blue point while the blue line represents the 95% PI. The orange dots represent the actual AUC as measured on the lesion

Figure 2 illustrates each lesion according to its ln-${\mathrm{SUV}}_{\mathrm{max}48}$ value as measured, and the ln-AUC as measured based on the four timepoints. As expected, a few data points fall outside the PI, but the PI covers the majority of lesions. To assess whether this prediction is accurate for lesions from new patients, the leave-one-out cross-validation was done for all 21 patients (Fig. 3). In all but 12 of the 208 lesions (6%, from 7/21 patients), the actual AUC based on four timepoints fell into the 95% PI. Based on the 48 h timepoint, our model shows good performance and demonstrates feasibility of using one timepoint to guide treatment decisions. Table 3 provides a useful statistical tool that allows treating clinicians to select a lesion within a patient that they wish to prescribe a radiation dose of at least 2000 cGy. The table columns provide the radioactivity amount that should be administered to have a 50%, 90%, 95%, and 97.5% probability of achieving a 2000-cGy target dose. For example, among lesions with a 48-h SUV_max_ of 10, 95% will have an AUC between 8.6 and 48.4. As a result, administration of 244.8 mCi will result in 50% of those lesions receiving at least 2000 cGy, while 431.4 mCi would result in 90% of those lesions receiving at least 2000 cGy. In patients with multiple lesions, those with higher SUVs at 48 h would be expected to receive proportionally higher doses, and those with lower SUVs lower doses. Based on the statistical model-derived prediction interval, Table 3 allows physicians to estimate the fraction of a patient’s lesion burden that will receive a given radiation dose such as 2000 cGy, which is expected to produce some therapeutic benefit, thereby assisting the physician in determining whether a patient will benefit from radioiodine therapy in most or some of the lesions.

**Table 3:** Prediction of AUC based on the 48 h SUV_max_ measured, and corresponding activity to be administered to deliver 2000 cGy

SUV_max_ at 48 h	AUC (uCi.h/g per mCi)		Activity to deliver 2000 cGy									
	Mean estimate	95% PI	To target 50% of the lesions		To target 90% of the lesions		To target 95% of the lesions		To target 97.5% of the lesions			
			(mean estimate)		(90% upper bound)		(95% upper bound)		(Upper bound of the 95% PI)		(Lower bound of the 95% PI)	
			GBq	mCi	GBq	mCi	GBq	mCi	GBq	mCi	GBq	mCi
2	4.0	1.5–9.1	45.4	1228.1	82.8	2236.5	96.7	2612.8	118.4	3200.4	20.1	542.4
4	8.1	3.3–20.3	22.7	613.2	39.5	1066.4	47.3	1278.0	54.6	1474.6	9.0	243.5
6	12.1	4.8–29.2	15.1	408.5	28.1	759.4	33.0	890.6	38.2	1032.0	6.2	168.9
8	16.1	6.4–38.8	11.3	306.2	21.1	570.5	25.6	692.4	28.7	775.6	4.7	127.4
10	20.2	8.6–48.4	9.1	244.8	16.0	431.4	19.1	517.0	21.3	576.6	3.8	102.1
15	30.3	11.9–71.0	6.0	163.1	10.8	292.3	13.2	355.5	15.4	416.2	2.6	69.6
20	40.4	16.1–97.1	4.5	122.2	8.1	217.6	9.4	254.8	11.4	306.9	1.9	50.9
30	60.6	24.3–147.4	3.0	81.4	5.7	153.1	6.6	179.1	7.5	202.9	1.2	33.5
50	101.2	40.8–252.9	1.8	48.8	3.3	87.9	3.8	102.7	4.5	121.1	0.7	19.5
100	202.6	82.3–479.6	0.9	24.4	1.7	45.3	2.0	53.1	2.2	60.0	0.4	10.3
200	405.8	164.2–951.2	0.5	12.2	0.8	21.5	1.0	26.0	1.1	30.1	0.2	5.2
300	609.2	257.8–1476.2	0.3	8.1	0.5	14.5	0.6	16.7	0.7	19.2	0.1	3.3

*SUV*, standardized uptake value; *AUC*, area under the curve; *uCI*, microCurie; *h*, hour; *g*, gram; *mCi*, millicurie; *cGy*, centiGray; *PI*, prediction interval.

Using the radioactivity concentration led to higher prediction error, while the use of SUL led to very similar prediction errors to those of SUV_max_ (Table 4). In addition, the prediction from other timepoints was reasonable but not as good as the 48-h timepoint, with CV prediction error for SUV_max_ of 0.472 at 24 h and 0.327 at 72 h versus 0.223 for a 48-h SUV_max_.

**Table 4:** Estimate of linear regression parameters, prediction error, and cross-validated prediction error, and estimated required activity to deliver 2000 cGy for different predictors using one timepoint

Timepoint	*N*	Slope	Robust se	Squared error	CV squared Error
uCi/g 24 h	231	1.018	0.050	0.615	0.665
uCi /g 48 h	231	0.934	0.043	0.443	0.484
uCi/g 72 h	231	0.859	0.051	0.679	0.761
SUV_max_ 24 h	217	1.057	0.045	0.436	0.472
**SUV**_**max**_** 48 h**	**208**	**1.002**	**0.024**	**0.204**	**0.223**
SUV_max_ 72 h	193	0.963	0.039	0.292	0.327
SUL 24 h	211	1.062	0.046	0.403	0.434
**SUL 48 h**	**200**	**1.013**	**0.028**	**0.207**	**0.225**
SUL 72 h	186	0.955	0.044	0.301	0.338

*uCi*, microCurie; *h*, hour; *se*, standard error; *CV*, cross-validated; *cGy*, centiGray. In bold: predictors with the lowest prediction error.

### Subset selected for high-dose RAI therapy

**Fig. 4:**
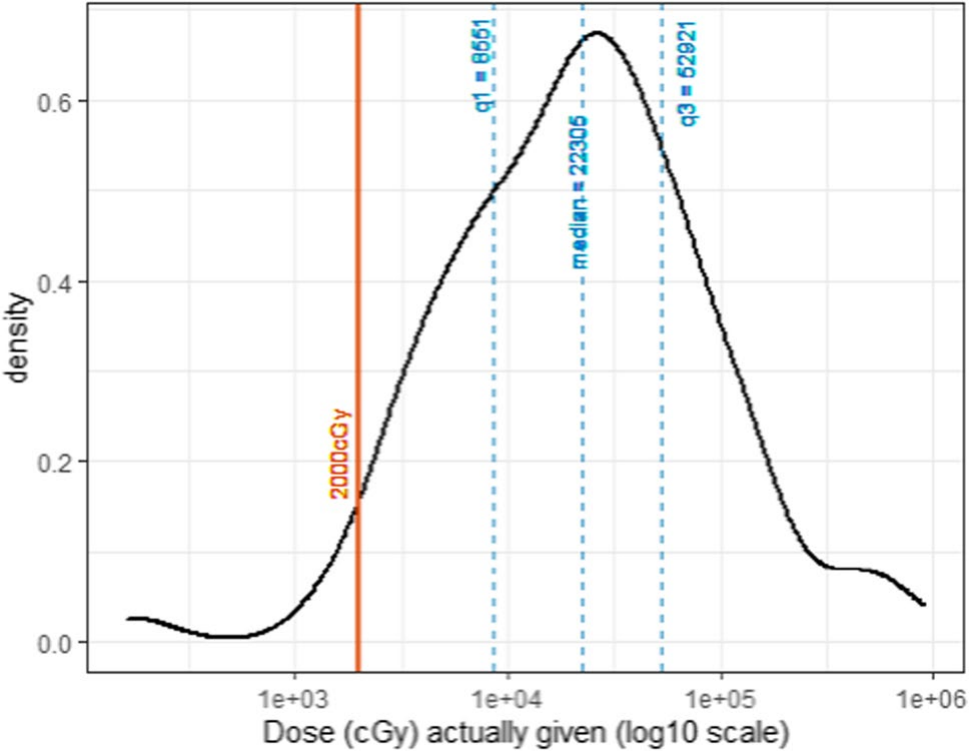
Maximum-intensity projection (MIP) PET ^124^I images at 48 h of 21 patents in the teaching set

**Fig. 5:**
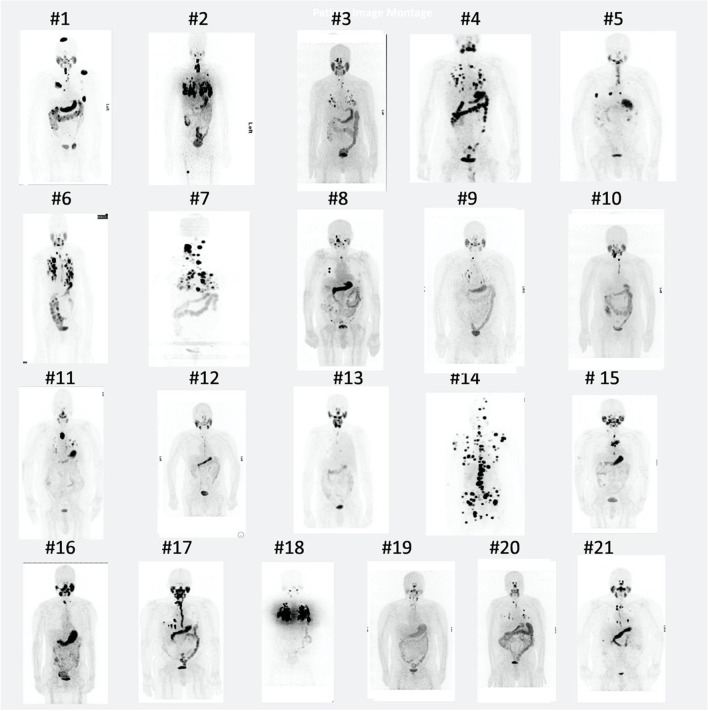
Distribution of radioactive iodine treatment dose given in 169 treated lesions (15 patients)

Patients were considered for RAI therapy when metastatic lesions showed active uptake of radiotracer predicted to be > 2000 cGy. A multidisciplinary tumor board reviewed the ^124^I lesional dosimetry data and in conjunction with other clinical parameters selected an administered activity that was at or below the MTA for each individual patient (Fig. 4). A subset of 15/21 patients representing 169/208 lesions subsequently underwent RAI therapy. The median lesion dose based on AUC was 22,305 cGy (interquartile range: 8551–52,921, range: 163–906,218 cGy; Fig. 5). Assuming reliable correspondence of near identical dose from ^131^I therapy as estimated from ^124^I PET dosimetry, we determined that of the 169 treated lesions, 163 (96%) received a dose greater than 2000 cGy. In the 14 patients treated and with negative thyroglobulin antibody level, all but 1 patient had reduction of thyroglobulin and 9/14 had greater than 50% reduction. The data described reflects the individual patient’s thyroglobulin nadir within the first 6 months post-treatment RAI. These findings provide an initial indication that predicted radiation-absorbed dose range of our treated population was consistent with efficacy in the majority of patients. Additional studies to verify these findings and provide details about dose response are pending.

## Discussion

In this manuscript, we report on the development of a dosimetry biomarker management approach to administer precision RAI therapy to patients with mDTC. The ^124^I PET imaging biomarker provides the treating physician with a tool to select the amount of radioactivity (mCi or MBq) expected to achieve a prescribed radiation-absorbed dose (cGy) to more than 97.5% lesions with SUVs above the selected value likely to achieve a therapeutic response. This manuscript focuses primarily on the biomarker method development, while more comprehensive validation studies with patient outcomes and dose–response findings in patients treated in an ongoing study will be discussed in subsequent manuscripts.

Like external beam radiotherapy, available data show that the treatment effectiveness of RAI at the individual lesion level is dependent on the radiation-absorbed dose to the individual lesion. Maxon et al. were among the first to make technically adequate quantitative dose estimates [19]. These measurements showed complete responses at 8500 cGy per lesion in 75% of metastatic thyroid cancer lesions to lymph nodes, and a treatment response threshold in a majority of lesions was observed at > 2000 cGy. Based on prior work by Maxon et al., we made an operational definition that a patient with any lesion with a predicted dose of > 2000 cGy would likely respond to treatment [19]. Therefore, in this study, we used an actionable threshold of 2000 cGy as the minimum radiation-absorbed dose for the patient to proceed with ^131^I RAI therapy, although other thresholds could be used. Consequently, mDTC patients are administered ^131^I RAI treatment only if they are likely to benefit from it.

As targeted therapy becomes more important to nuclear medicine, there is growing recognition of the need for quantitative dosimetry and an emphasis on identifying the relationship between radiation-absorbed dose in cGy and tumor response. In this regard, classic studies by Maxon provided benchmarks for cGy dose for complete response, for two types of ^131^I-avid tissues: thyroid remnant (~ 30,000 cGy) and metastatic well-differentiated thyroid cancer, to cervical lymph nodes (~ 8500 cGy) [18]. Applications using the ^124^I-^131^I theranostic pair have further improved knowledge of radiation-absorbed dose and treatment response. Jentzen et al. [20] reported pioneering applications of ^124^I as a theranostic surrogate to ^131^I in mDTC and confirmed the general conclusions of Maxon et al with regard to dose response for normal thyroid remnants and mDTC in lymph nodes. These investigators studied 34 patients with 227 lesions in a systematic way with ^124^I imaging before and after RAI therapy. They found two classes of lesions and defined them as > 0.8 ml or < 0.8 ml. This threshold was related to the resolution of PET imaging used to identify sites of uptake. For larger lesions, it was possible to define lesion volume using CT reasonably accurately; for smaller lesions, the resolution volume of 0.8 ml was assumed and thus a “minimum” estimate of cGy could be determined. All 57 large-volume lesions were treated to > 8500 cGy when possible and response rates were highest for pulmonary metastases (88%), lower for lymph nodes (63%), and lowest for bone metastases (50%). Individual lesion-absorbed dose estimates were made using two imaging time points (24 h and 4 days) [3]. Overall, the 168 smaller lesions had a significantly higher response rate of 82–88% for lymph nodes; for pulmonary metastases, the response rate was 100%, with radiation dose estimates varying from 1200 to 10,000 cGy. Toxicity was controlled by planning the amount of MBq administered to ensure that cGy to blood was < 200 cGy.

Using the proposed approach, we confront a major problem of RAI therapy: the considerable heterogeneity of radiation-absorbed dose to lesions within a given patient, and between patients with mDTC, at a given amount of MBq ^131^I administered. Variation in measured cGy dose from lesion to lesion may be both technical and biologic in nature. Although the technical features such as difficulty in imaging small tumors quantitatively may play a role in inaccurate dosimetry, it is likely that the observed differences in cGy from lesion to lesion is predominantly biological in nature. This hypothesis is being actively explored.

There was a marked heterogeneity of AUC values in those lesions as well as a broad range of SUV_max_ at 48 h, ranging from 1 to 983. The AUC is the measured parameter that can be used to compute radiation-absorbed dose for individual lesions and so as expected, when this is done according to the methods described above, the dose in cGy can be determined. In our treated group of 15 patients, the patients had an average of 12 lesions (range 4–23) and these had a wide variability in AUC and therefore radiation-absorbed dose. However, we can focus on the lesion with the lowest SUV_max_ at the 48-h timepoint. We can then define the MBq amount of RAI that will give this lesion at least 2000 cGy. If we target the lesion with the lowest uptake with enough ^131^I to have a prescribed probability of getting > 2000 cGy, then we assume that all other lesions in this patient will have at least this radiation-absorbed dose, and therefore, the patient is likely to respond to this effective dose. Thus, we have personalized the dose to be optimal for this particular patient.

This calculation can be thought of as precision therapy, because it provides a statistical estimate of the probability that the target lesion dose will be achieved. We can prescribe this absorbed dose as long as our MTA activity is equal to or greater than the calculated activity needed to cover a chosen fraction of lesions (e.g., 95% of all lesions with a prescribed dose rate). If the needed activity to target 95% of the lesions is above the MTA, a lower activity can be chosen if targeting a lower but still desirable portion of the patient’s lesions. In this work, we selected 2000 cGy as the radiation-absorbed dose threshold for an effective treatment, but the proposed approach can easily be adapted for any other threshold value deemed necessary.

When investigating a single timepoint predictor, the 48-h timepoint was found to be the best single time-point predictor of the average integrated AUC uptake (and thereby the radiation-absorbed dose) for individual lesions. Further research is warranted to explore the impact of characteristics such as clearance, as it can vary greatly from one patient to another, and to encapsulate outlier radioiodine kinetic profiles into the prediction model. This will include extending the regression model to the possibility of adding a second timepoint for the prediction. The current research incorporates useful information about the variability in lesion uptake by considering all lesions from all subjects in the calculation of a prediction interval, in order to best determine the predicted prescribed radioactivity to achieve a radiation-absorbed dose that exceeds the desired threshold for therapeutic efficacy with a stipulated precision, typically 90% or 95% probability. Note that SUV_max_ was used to represent uptake, as only a small fraction of the lesions were large enough to observe heterogeneity of the radioiodine distribution.

Our approach shows promising results in demonstrating a correlation between integrated AUC and a single timepoint in our learning set of 21 patients, but to further improve the precision of our predictor, recruitment of a larger patient cohort is in process. A simulation study estimated that an increase in sample size from 21 to 60 patients would increase the precision (as measured by the half-width of the 95% PI on the log-scale) from 1.38 to 1.33, but beyond this number, the gain is very small (1.31 and 1.30 with 120 and 1000 patients, respectively).

Other investigators have also recognized this need for a practical single-timepoint imaging method, particularly to assure the patient safety of those undergoing theranostic treatments [21-35]. Hänscheid et al. investigated the accuracy of a single imaging timepoint to predict the dosimetry for key normal tissues and tumor vs. clearance fitting from serial gamma camera images from ^177^Lu-DOTATATE or ^177^Lu-DOTATOC treatments [33]. In that study, they looked at the dose to kidney, liver, spleen, and 30 NET lesions following the administration of ^177^Lu-DOTATATE or ^177^Lu-DOTATOC. They studied different timepoints post-administration and found the lowest maximum errors at 96 h and reported deviations from the time integral of median of + 5% (range, − 9 to + 17%) for kidneys, + 6% (range, − 7 to + 12%) for livers, + 8% (range, + 2 to + 20%) for spleens, and + 6% (range, − 11 to + 16%) for NET lesions [19]. Willowson et al. performed a similar study with a focus on kidney dosimetry to anticipate renal toxicity [23]. They reported an average deviation from doses obtained from complete image data on cycle 1 of 13% and 2% when using 4-h data only and 24-h data only. A recent study by Hou et al. [29] examined different theranostic agents and suggested that simplified single-timepoint dosimetry approaches may work well for ^177^Lu-DOTATATE, but the generalizability of single-timepoint imaging for dosimetry for certain targeting agents such as ^177^Lu-PSMA targeted bone metastases may be less successful.

We believe that any dosimetry method used as a surrogate for an actual radionuclide therapy must be proven to be of value in predicting treatment response. In the case of ^124^I for ^131^I-RAI, the issue of differences in tissue and tumor radiotracer kinetics between the ^124^I radiotracer distribution and the ^131^I therapy administration remains incompletely studied. However, they are exact isotopic substitutions and therefore can be assumed to exhibit identical tissue kinetics for the purposes of performing dosimetry. In the present paper, we talk primarily about methods and approaches, rather than outcomes and validation. However, based on these concerns we have remarked upon very preliminary but relevant data on TG within 6 months in support of the concept that ^124^I dosimetry may be a useful surrogate for ^131^I-RAI (data planned for more complete presentation in later publications). Also, we document that measured TG responses were observed in those patients for whom the computed dosimetry profile resulted in lesions receiving radiation-absorbed doses > 2000 cGy (with some higher uptake lesions receiving > 8500 cGy) given ^131^I activities consistent with dose-limiting toxicity constraints.

Finally, personalized radioiodine dosimetry in RAI focused on estimating the MTA would ensure that treatment would not result in a blood and whole-body dose that would exceed the threshold for serious bone suppression or radiation lung fibrosis for patients with extensive lung metastases [18]. The shift in dosimetry emphasis being proposed here is toward the rational selection of treatment activity based on a population-averaged statistical model relating single-timepoint ^124^I lesion SUV measurements with dose expectation and subsequently response prognosis, consistent with the normal tissue-limiting MTA.

Clinically, we recognize that quantitative SPECT imaging is an alternative approach to lesion dosimetry. Since ^131^I is clinically approved and widely used, potentially developing a single time point approach to lesion dosimetry based on ^131^I is certainly appealing, but the technical limitations of SPECT would greatly limit the range of lesions adequately assessed to the minority of lesions, to the larger lesions. Moreover, ^124^I PET has major technical advantages mainly related to a combination of higher sensitivity (50–300 times) and better resolution, which translates into significantly better quantitative performance, especially for small metastatic lesions [36, 37]. For these reasons, we chose PET and ^124^I for the proof-of-principal phase of biomarker development and in the setting of clinical research.

In summary, we have provided initial validation of a single time point lesion dosimetry biomarker utilizing ^124^I PET scanning. Our study population had macroscopic lesions and so our approach to determining appropriate choice of amount of ^131^I for a given predicted cGy dose is designed for this clinical situation. When coupled with a knowledge of the MTA determined by blood and whole-body clearance, clinicians can utilize the relationship between administered activity and lesion dosimetry to optimize a RAI treatment strategy that maximizes therapeutic effectiveness while minimizing the risk of serious adverse events. Whereas not a requirement in all countries, Directive 2013/59, article 56 of the European Union, mandates dosimetry for radionuclide therapy optimization by law, and therefore, methods to facilitate such approaches can be an important addition to the field [29].The intent of this manuscript is to provide a statistical analysis of the precision with which one can estimate lesion dosimetry under the constraint of a single imaging time point, while recognizing that two or more imaging time points will improve lesion-absorbed dose estimation.

## Supplementary Information

Below is the link to the electronic supplementary material.

Supplementary file1 (DOCX 16.5 KB)

Supplementary file2 (DOCX 16.6 KB)

## Data Availability

Data used in the analysis are provided in the Supplementary Material.
