# Supplementary material for: The use of single-timepoint images to link administered radioiodine activity (MBq) to a prescribed lesion radiation-absorbed dose (cGy): a regression-based prediction interval tool for the management of well-differentiated thyroid cancer patients
Source: Eur J Nucl Med Mol Imaging. 2023 May 12;50(10):2971–83. doi: 10.1007/s00259-023-06240-1 (PMC10382352; doi:10.1007/s00259-023-06240-1)
Supplement: Supplementary file 1 — Supplementary file1 (DOCX 16.5 KB) [file 259_2023_6240_MOESM1_ESM.docx]

**Supplementary Data: Appendix A**

**Building a Prediction Interval**

The approach used is the one presented in Gelman and Hill (2007; page 272) to make prediction for a new lesion in a new patient.

Let $i=1, \ldots, n$ indicate the patients included in the training set $\mathbb{T}$, and $j=1, \ldots, n_{i}$ indicate the lesions. The expectation of the log(AUC) for the lesion $j$ of patient $i$ is denoted by $E\left( y_{ij} \right)= \mu_{ij}$ while $x_{ij}$ is the uptake measured at one time-point, eg the logarithm of 48h SUV measured on lesion $j$ from patient $i$.

The linear model is defined as:

$$y_{ij}= {x'}_{ij}\beta+ \varepsilon_{ij}= \mu_{ij}+ \varepsilon_{ij}$$

The error terms $\varepsilon_{ij}$ are correlated, and generalized estimating equations (GEE) are used for the estimation (Zeger et Liang 1986) assuming an exchangeable correlation structure.

To predict the value $\tilde{y}_{i1}$ for a new lesion in a new patient $p \left( p\notin\mathbb{T} \right)$ with value $\tilde{x}_{i1}$, we need to generate a new error $\varepsilon_{i1}$ for this lesion, then calculate the predicted data. The detailed steps are as follows:

1. Estimate values for $\hat{\beta}$, the covariance matrix $V_{\beta}$ and the residual variance $\hat{\sigma}^{2}$ using the linear model.
2. Draw values for $\sigma_{s}^{2}$ using $\sigma_{s}= \hat{\sigma} \sqrt{\left( n-k \right)/ \chi}$, where $k$ is the number of predictors, and $\chi$ is a random draw from a $\chi^{2}$ distribution with $n-k$ degrees of freedom.
3. Draw values for $\beta_{s}$ from a multivariate normal distribution with mean $\hat{\beta}$ and variance $\sigma_{s}^{2}V_{\beta}$.
4. Draw values for $\tilde{y}_{i1}$ from a multivariate normal distribution, with mean $\tilde{x}_{i1}\beta_{s}$ and variance $\sigma_{s}^{2}$.

A total of 1000 simulations were used. The mean of the 1000 simulated values is used as the predicted value, while the 2.5% and 97.5% percentiles are used to define the lower and upper boundaries 95% confidence interval. Other percentiles, such as 95% or 90%, can also be used for prediction purpose.

**References**

Gelman A and Hill J. Data Analysis Using Regression and Multilevel/Hierarchical Models. Cambridge University Press, 2007.

Zeger SL and Liang K. “Longitudinal Data Analysis for Discrete and Continuous Outcomes.” Biometrics 42, no. 1 (1986): 121–30.
