# Supplementary material for: The use of single-timepoint images to link administered radioiodine activity (MBq) to a prescribed lesion radiation-absorbed dose (cGy): a regression-based prediction interval tool for the management of well-differentiated thyroid cancer patients
Source: Eur J Nucl Med Mol Imaging. 2023 May 12;50(10):2971–83. doi: 10.1007/s00259-023-06240-1 (PMC10382352; doi:10.1007/s00259-023-06240-1)
Supplement: Supplementary file 2 — Supplementary file2 (DOCX 16.6 KB) [file 259_2023_6240_MOESM2_ESM.docx]

**Supplemental Table 1**: Patient Dosimetry Summary

| Patient | Lesion # | Size (cm) | Teff (d) | SUV(48h) | Activity to give 20Gy (mCi) | Admin Activity (mCi) | Dose (Gy) |
| --- | --- | --- | --- | --- | --- | --- | --- |
| 1 | 13 | 1.9 (0.4-4.27) | 8.02 (2.21-8.02) | 8.03 (1.24-127) | 72.1 (8.51-599.7) | 407 | 112 (13.6-955) |
| 2 | 18 | 0.52 (0.22-2.45) | 4.28 (0.78-8.02) | 80.1 (0.7-983) | 3.0 ( 0.43-1132) | 194 | 1,285 (3.4-9,062) |
| 3 | 14 | 0.87 (0.63-1.97) | 0.45 (0.4-7.42) | 3.25 (0.6-11.9) | 135 (30.2-351) | Not Treated | N/A |
| 4 | 12 | 0.68 (0.30-1.67) | 3.16 (1.31 - 7.89) | 7.4 (3.6-26.5) | 61.3 (10.95-165.3) | 222.7 | 73 (26.9-406) |
| 5 | 10 | 0.72 (0.30-2.47) | 1.48 (1.01-3.57) | 1.85 (0.50-26.4) | 126 (12.7-1451) | Not Treated | N/A |
| 6 | 16 | 0.93 (0.60-4.37) | 1.59 (0.43-4.38) | 27.8 (0.50-119) | 26.38 (2.84-2959) | 196.8 | 151 (1.3-1387) |
| 7 | 23 | 0.97 (0.30-1.97) | 2.48 (0.99-7.89) | 35 (1.0-783) | 20.4 (1.06-1854) | 236.1 | 232 (18.5-122631) |
| 8 | 6 | 0.67 (0.23-1.1) | 4.41 (1.74-6.29) | 1.73 (0-36.6) | ND (74.1-infinity) | 138.2 | 18.6 (0-273) |
| 9 | 8 | 0.58 (0.43-1.27) | 3.03 (1.1-7.14) | 4.35 (1.6-11.5) | 99.3 (61.7-435) | Not Treated | N/A |
| 10 | 4 | 2.37 (1.7-4.13) | 1.26 (0.92-2.29) | 26.2 (0.70-515) | 9.87 (0.034-24.96) | 158.4 | 49.2 (1.27-930) |
| 11 | 11 | 0.40 (0.30-2.80) | 2.98 (1.47-4.06) | 11.30 (6.9-189) | 15.37 (4.11-59.2) | 171.4 | 223 (57.9-834) |
| 12 | 3 | 0.87 (0.30-1.30) | 3.8 (0.52-7.89) | 0.7 (0.50-5.3) | 179.9 (71.8-746) | Not Treated | N/A |
| 13 | 13 | 0.90 (0.43-3.43) | 1.55 (0.86-8.02) | 1.30 (0.50-4.2) | 1175 (46.9-1695) | Not Treated | N/A |
| 14 | 5 | 0.30 (0.23-1.13) | 1.74 (0.54-1.85) | 0.90 (0.30-3.20) | 395 (69.1-2026) | Not Treated | N/A |
| 15 | 17 | 1.57 (0.77-2.37) | 3.79 (1.67-8.02) | 385.3 (13.5-707) | 2.47 (1.24-27.59) | 46.08 | 372 (33.4-744) |
| 16 | 6 | 1.22 (0.60-3.83) | 4.77 (2.29-8.02) | 8.30 (2.6-20.3) | 99.7 (50.7-140.9) | 220.8 | 44.3 (31.3-87.1) |
| 17 | 6 | 0.58 (0.27-0.77) | 3.04 (1.22-8.02) | 0.80 (0-4.80) | ND (91.8-infinity) | 392.6 | 21.15 (0-85.5) |
| 18 | 12 | 0.35 (0.27-0.97) | 1.69 (1.22-2.86) | 25.1 (3.1-78.0) | 10.97 (2.27-45.3) | 147.9 | 272 (65.4-1306) |
| 19 | 17 | 0.83 (0.40-2.0) | 1.55 (1.09-6.43) | 204.6 (76.5-629) | 3.70 (0.93-10.27) | 98 | 529 (192-2096) |
| 20 | 13 | 0.50 (0.20-1.97) | 1.74 (1.22-2.81) | 2.30 (0.50-15.3) | 65.1 (21.9-1935) | 239 | 73.4 (2.47-218.4) |
| 21 | 10 | 0.68 (0.43-1.50) | 4.63 (2.46-8.02) | 10.8 (1.1-21.7) | 77.3 (14.75-295) | 398.4 | 104.1 (26.99-540) |

Column 6 & 7 - to change the activity units to GBq multiple by 0.037.
